# Prevalence and risk factors for red blood cell alloimmunisation among sickle cell patients in Mwanza City, Tanzania

**DOI:** 10.4102/ajlm.v9i1.823

**Published:** 2020-09-10

**Authors:** Erius Tebuka, Mwesige Charles, Jeffer O. Bhuko

**Affiliations:** 1 Department of Pathology, Weill Bugando School of Medicine, Mwanza, United Republic of Tanzania; 2 Bugando Medical Centre, Mwanza, United Republic of Tanzania; 3 Central Pathology Laboratory, Bugando Medical Centre, Department of Hematology, Mwanza, United Republic of Tanzania; 4 Mwanza Region Health Center, Mwanza, United Republic of Tanzania

**Keywords:** sickle cell disease, alloimmunisation, alloantibody, screening cells, Bugando Medical Centre, Catholic University of Health and Allied Sciences, red blood cells

## Abstract

**Background:**

Erythrocyte alloimmunisation can lead to complications such as delayed haemolytic transfusion reaction.

**Objective:**

This study investigated the prevalence of and risk factors for red blood cell alloimmunisation among multiply transfused sickle cell disease (SCD) patients in Mwanza City, Tanzania.

**Methods:**

From May 2017 to July 2017, this descriptive, cross-sectional, hospital-based study enrolled 200 participants with SCD who had received at least two units of blood in the previous year. Blood count was performed using a Sysmex haematology analyser. Antibody screening was done by the tube method using a panel of three screening cells with known antigenicity.

**Results:**

Of the 200 patients enrolled, 108 (54%) were female. The median age was 4.5 years (interquartile range [IQR] = 6), the median number of transfusions was 3 (IQR = 1), and the median pre-transfusion haemoglobin level was 6.6 g/dl (IQR = 2.7). Prevalence of alloimmunisation was 8.5% (17/200) with immunoglobulin G, and one patient developed cold immunoglobulin M antibodies. Blood groups reported were Rhesus C and E, Kell, Kidd and Duffy. There was no statistically significant association between the number of transfusions and the risk of alloimmunisation.

**Conclusion:**

The rate of alloimmunisation in multiply transfused SCD patients was 8.5% and higher than other studies in East Africa. Thus, there is a need for extensive red blood cell screening and matching to minimize alloimmunisation and risk of delayed haemolytic transfusion reaction, particularly in SCD and chronically transfused patients.

## Introduction

Sickle cell disease (SCD) is an inherited haemoglobin disorder; it is a genetic disease with high prevalence in the equatorial regions of Africa, Arabia, Europe, and India. Globally, approximately 300 000 people are born with SCD annually.^1^

Blood transfusions are necessary for the care and treatment of patients with SCD.^2,3^ In Africa, including Tanzania, and the Middle East, the ABO and Rhesus D blood grouping system are as the test parameters for blood transfusion, neglecting other red blood cell surface antigens, which are considered minority blood groups. However, this can cause serious complications, including alloimmunisation in SCD patients, once a transfusion is done, without a thorough and complete screening of the antigenicity of both the donor and the recipient.^4^

Alloimmunisation is the body’s response to foreign antigens after being subjected to cells or tissues with different antigenicity.^5^ Complications of alloimmunisation include life-threatening conditions such as delayed hemolytic transfusion reactions, immediate agglutination, hyper hemolysis^2^ and autoimmune responses in individuals having blood transfusions, especially SCD patients.

Studies have shown that antigenicity matching, for SCD and chronically transfused patients, is important for decreasing the frequency of alloimmunisation and its associated complications^6^. The presence of alloantibodies is a major complication in SCD, presenting challenges for medical management. Antigen matching beyond the standard ABO blood grouping system and Rhesus typing has reduced complication rates.^6^ According to Bashawri et al., race and ethnicity could be a risk factor for alloimmunisation in sickle cell anaemia patients.^7^ Additionally, failure to screen for minor antigens such as Kidd, Duffy, and MNS blood groups, might cause a mismatch and subsequent alloimmunisation in blood recipients.^8^.

Current practice in Tanzania is forward grouping, which screens for blood groups ABO and Rhesus D. However, there are currently no guidelines for blood group screening at the National Blood Transfusion Services. Thus, patients are typically transfused without an extensive matching for compatibility. For example, no screening for Kell antigens is conducted. Additionally, little is known about the extent to which alloantibodies affect sickle cell patients locally.

This study aimed to examine the prevalence of developing red blood cell alloimmunisation among multiply transfused SCD patients and identify its associated risk factors, especially in Mwanza (Lake Zone). This will, in turn, be valuable for the setting up of guidelines for extensive donor and recipient typing, especially for transfusion-dependent patients at Bugando Medical Centre (BMC)/Lake Zone National Blood Transfusion Services laboratories.

## Methods

### Ethical considerations

Ethical clearance was received from the Catholic University of Health and Allied Sciences and BMC joint research committee (ethical clearance number: 323/2017). Permission to work in the BMC Central Pathology Laboratory was also sought from the Laboratory Director and Manager. Written informed consent was sought from both inpatient and outpatient SCD with multiple transfusions before including them in the study.

### Study design

This was a cross-sectional study conducted from May 2017 to July 2017 at the BMC, Mwanza City (Lake Zone), Tanzania. Participants were inpatients and outpatients with SCD who attended the BMC during the study period and consented to participate. Patients were recruited until the estimated minimum sample size of 200 SCD patients was reached; the sample size was estimated using the Kishi Lisle Formula. The study included all SCD patients attending BMC inpatient and outpatient departments for transfusion therapy purposes who had received at least two units of blood during the previous year The study excluded patients with no blood transfusion history and patients who did not consent to participate. A structured questionnaire was completed by the parent or guardian of the child and laboratory records confirmed SCD, number of transfusions and interval of blood transfusion. The questionnaire was used to gather the demographic and medical history information of selected participants.

### Sample collection

One 4-ml blood sample was collected from each patient by phlebotomists at the Central Pathology Laboratory at BMC into ethyldiammine tetraacetate tubes to avoid agglutination and preserve red blood cell (RBC) antigenicity. Samples were stored at 2 °C – 6 °C refrigeration for not more than 3 days.

### Laboratory analysis

#### Alloantiboy detection – Indirect Coombs test

The presence of alloantibodies was determined by assessing antigen-antibody agglutination in patient serum where agglutination indicates incompatibility of patient’s serum antibodies with commercially purchased red cells. Three commercially purchased red cell-antibody screening cell panels were used for screening the parted blood cells (screen cell 1, product code R_1_^W^R_1_; screen cell 2, product code R_2_R_2_; and screen cell 3, product code rr) (Lorne Laboratories, Reading, Berkshire, United Kingdom). Screening for the different RBCs antigens was done as described by the manufacturer. Screen cell 1 had C, D, e, C^w^, M, N, s, P1, K, k, Le^b^, Fy^a^, Jk^a^, and Jk^b^ red cells. Screen cell 2 had D, E, c, M, S, P1, k, Le^b^, Fy^b^, and Jk^b^ red cells, and screen cell 3 had c, e, N, s, P1, k, Kp^a^, Le^a^, Fy^b^, and Jk^a^ red cells (Table 1). A mixture of patient serum and the commercial red cell-antibody screening cells was centrifuged for 10 minutes at room temperature (25 °C). Tubes were checked for agglutination confirming cold alloantibodies (immunoglobulin M). Then Low ionic strength solution was added to enhance antigen-antibody reaction, followed by incubation at 37 °C for 15 min to detect warm antibodies. After incubation, a cell wash was done by using a washing buffer to remove unbounded antibodies. Finally, anti-human globulin (Coombs reagent) was added to the tubes to detect the presence of alloantibodies. The tubes were then centrifuged at 1500 revolutions per minute for 3 min. The centrifuged mixture of patient serum, commercial red cells and anti-human globulin was read microscopically to assess the presence of agglutination. Tubes with agglutination were considered ‘positive’, whereas tubes with no agglutination were considered ‘negative’. Agglutination was classified as ‘strong’ when agglutination could be seen macroscopically or as ‘mild’ when agglutination could only be confirmed microscopically. Alloantibodies were then classified as ‘warm’ alloantibodies (antibodies that react at or near 37 °C) or as ‘cold’ alloantibodies (antibodies that react below 37 °C).

**TABLE 1:** Screening cells used and blood groups tested for alloantibodies, Bugando Medical Centre, May 2017–June 2017.†

Screening cell panel	Blood group																			
	Rhesus						MNS				Kell			P1	Lewis		Duffy		Kidd	
	C	E	c	e	D	C^w^	M	N	S	s	K	k	Kp^a^		Le^a^	Le^b^	Fy^a^	Fy^b^	Jk^a^	Jk^b^
Screen 1 (R_1_^W^R_1_)	+	0	0	+	+	+	+	+	0	+	+	+	0	+	0	+	+	0	+	+
Screen 2 (R_2_R_2_)	0	+	+	0	+	0	+	0	+	0	0	+	0	+	0	+	0	+	0	+
Screen 3 (rr)	0	0	+	+	0	0	0	+	0	+	0	+	+	+	+	0	0	+	+	0

† , Information as provided from manufacturer of the screening cells, Lorne Laboratories (Reading, Berkshire, United Kingdom).

#### Autoantibody detection – direct antiglobulin test

To rule out autoantibodies, which were not part of the study but can affect test results, a direct antiglobulin test was done. Briefly, the patient’s red blood cells were washed three times with normal saline. Two drops of anti-human globulin (Coombs reagent) was added to the washed cells, mixed and centrifuged at 1500 revolutions per minute for 3 min. The centrifuged mixture of patient RBC and anti-human globulin was read microscopically for agglutination. Tubes with agglutination were considered ‘positive’ for autoantibodies, whereas tubes with no agglutination were considered ‘negative’ for autoantibodies. To rule out anti-human globulin non-reactivity and validate negative results (tubes without agglutination), pre-sensitised commercial RBCs (Lorne Laboratories; Reading, Berkshire, United Kingdom) coated with short-armed antibodies were added. If agglutination was observed, which was expected, then anti-human globulin was reactive and the patient’s result was considered valid.

### Data management and statistical analysis

After cross-checking, data were transferred directly to Statistical Package for Social Sciences software (International Business Machines Corporation, Chicago, Illinois, United States) version 20 and analysed. Continuous data were presented using medians and interquartile range, and the Chi-square was used to test associations for categories available. *P* < 0.05 was considered statistically significant.

## Results

The median age of the study participants was 4.5 years (range: 0–26 years) (Table 2). Of the 200 patients enrolled, 17 patients were positive for alloantibodies. No autoantibodies were detected in any patient; all direct antiglobulin tests were negative. One patient developed immunoglobulin M cold antibodies and was excluded from downstream analysis, but did not experience the consequent nuisance hemolysis.

**TABLE 2:** Demographic characteristics of sickle cell disease patients with multiple transfusions at Bugando Medical Centre, May 2017–July 2017.†

Variable	Median		%
	IQR	Frequency	
Number of transfusions received	3.00	1	-
Interval between transfusions (days)	0.50	1.5	-
Pre-transfusion haemoglobin (g/dl)	6.40	3.7	-
**Sex**			
Female	92	-	46
Male	108	-	54
**Age (years)**			
Median	4.50	6	-
0–5	114	-	57.0
6–12	54	-	27.0
13–26	32	-	16.0
**Number of transfusion units**			
2 units	99	-	49.5
3–4 units	78	-	39.0
≥5 units	23	-	11.5
**Number of admissions**			
Inpatients			
One admission	68	-	34.0
Two or more admissions	74	-	37.0
**Outpatients**			
One admission	23	-	11.5
Two or more admissions	35	-	17.5
**Interval between blood transfusions (days)**			
Less than 1 day	97	-	48.5
One day	69	-	34.5
More than one day	34	-	17.0
**Parents’ marital status**			
Single	79	-	39.5
Married	121	-	60.5
**Parents’ occupation**			
Unemployed	24	-	12.0
Employed	95	-	47.5
Peasants/business	81	-	40.5
**Warm IgG (%)**			
Yes	17	-	8.5
No	183	-	-
**Cold IgM (%)**			
Yes	1	-	0.5
No	-	-	-

IgG, immunoglobulin G; IgM, immunoglobulin M; IQR, interquartile range.

† , *N* = 200 sickle cell patients.

A total of 17 (8.5%) patients developed 23 warm alloantibodies and 183 (91.5%) tested negative for warm alloantibodies (Table 3). Of the 17 patients that developed warm alloantibodies, 11 had single alloantibodies and six had double alloantibodies. Of the 23 identified alloantibodies, 87% (20/23) showed mild agglutination, and 13% (3/20) showed strong agglutination. Among the alloimmunised patients, the number of transfusions ranged from 3 units to 6 units within the previous year. There was no significant statistical association between the number of transfusions and the risk of alloimmunisation [*p* = 0.07]. The median number of transfusions for patients without alloantibodies was 2 (median: 1.0) units of blood. All 183 patients who were negative for warm alloantibodies received ≤ 2 transfusions.

**TABLE 3:** Demographic characteristics of sickle cell patients having warm alloantibodies at Bugando Medical Centre, May 2017–July 2017.

Variable	Warm IAT test results (IgG)				*P*
	Positive		Negative		
	*N*	%	*N*	%	
**Patients**	17	8.5	183	91.5	-
**Sex**					
Male	10	58.8	98	53.6	0.528
Female	7	41.2	85	46.4	-
**Age (years; median)**	4.7	3.9	4.5	6.0	0.355
**Number of transfusions (median)**	3.5	4.0	2.0	1.0	0.070
**CBC indices**					
Haemoglobin	7.1	-	6.3	-	-
MCV	78.0	-	79.2	-	-
MCHC	31.0	-	31.2	-	-
RDW	18.1	-	18.1	-	-
Total WBC	6.7	-	9.5	-	-
Neutrophils	2.93	-	4.18	-	-
Lymphocyte	2.01	-	3.42	-	-
Thrombocyte	233	-	212	-	-
**Number of admissions (median)**	1.0	-	1.0	-	0.836
**RhesusD screening**					
Positive	17.0	8.5	183	91.5	NA
Negative	0.0	-	0	-	-
**Post transfusion haemoglobin (g/dl)**					
Rise	7.0	58.3	100	68.2	0.596
Fall	5.0	41.7	50	31.8	-
**Interval between transfusions (days)**	0.5	0.5	0.5	1.5	0.184

BMC, Bugando Medical Centre; BT, blood transfusion; CBC, cell blood count; IAT, indirect antiglobulin test; IgG, immunoglobulin G; MCHC, mean cell haemoglobin concentration; MCV, mean cell volume; NA, not applicable; RDW, red cell distribution width; RH, Rhesus; SCD, sickle cell disease; WBC, white blood cell.

Of the 23 identified alloantibodies, 12 were of the Rhesus group (anti-E [2], anti-C [6], anti-c [3], anti-e [1]), 6 were of the Kell group (anti-K), 2 were of the MNS group (anti-M), 1 was of the Kidd group (Jk^a^), 1 was of the Duffy group (Fy^a^) and 1 was of the Lewis group (Le^a^) (Table 4).

**TABLE 4:** Warm alloantibodies in sickle cell patients with multiple blood transfusions who tested positive for alloantibody screening, Bugando Medical Centre, May 2017–June 2017.

Antibody†	Patient Number‡	Frequency (%)
**Rh system**		
**Ant-C, Ant-E**	9	52.94
**Ant-c, Ant-e**		
Ant-D, Ant-C^w^		
**Kell system**		
Anti-K	3	17.64
**MNS system**		
Ant-M, Ant-N	2	11.76
Ant-S, Ant-s
**Duffy system**		
Fy^a^, Fy^b^	1	5.88
**Kidd system**		
Jk^a^, Jk^b^	1	5.88
**Lewis system**		
Le^a^, Le^b^	1	5.88
**P system**		
P1	0	0.00

† , refer to red blood cell antigens that were combined, for example, Fy^b^, Kp^a^, Fy^a^.

‡ , A total of 17 patients developed 23 alloantibodies; some individuals were positive for more than one alloantibody.

## Discussion

The alloimmunisation rate in the current study is higher than the 6.1% alloimmunisation rate reported by Natukunda et al. in Mulago Kampala Uganda and the 4.1% rate reported by Meda et al. in Muhimbili Dar es salaam, Tanzania.^9,10^ The differences in these rates could be attributed to differences in the median number of blood transfusions, which was lower (mean, 2(0) units) in the other two studies, whereas the mean number of transfusions at BMC was 3(1) units. The alloimmunisation rate of the current study was comparable to that reported by Ugwe et al.^3^ of 9.3%. The study included 145 SCD patients in Nigeria; blood transfusion was found to be significantly associated with alloimmunisation (*p* = 0.027 where *p* < 0.05). As with our study, sex was not statistically significantly associated with the risk of alloimmunisation in all three studies.

As of 2018, Tanzania was the third leading country in Africa for sickle cell anaemia after the Democratic Republic of the Congo and Nigeria.^11^ The detection of alloantibodies of the Kell, MNS, Kidd, Duffy and Lewis blood groups is common^12^ and is corroborated by our study as Anti-C, anti-c, Anti-E, anti-e, Anti-K were the most common alloantibodies detected. Thus, these RBC antigens should be included in extended blood typing to detect alloantibodies to these groups. Warm alloantibodies (often IgG) are of clinical importance because they haemolyse red blood cells at body temperature whereas cold antibodies (IgM) also known as nuisance antibodies rarely haemolyse red blood cells *in vivo* due to temperature inactivation.^13^

In Tanzania, the Lake Zone area is leading with SCD patients (BMC, Mwanza) hence the numerous transfusions as part of their clinical management^14^. Thus, the prevalence and risk factors for red blood cell alloimmunisation in this locale needs to be identified. This study found an alloimmunisation rate of 8.5% among SCD patients at BMC in Mwanza, Tanzania, but found no significant association between alloimmunisation and the increased number of transfusions.

### Limitations

Multiple transfusion is a risk factor for alloimmunisation, especially in sickle cell disease patients. However, in our study, multiply transfusion was not significantly associated with alloantibody development. This could be, in part, due to the small sample size, under-reporting and maybe because all participants had received blood transfusion at least once. More than half of the participants’ guardians or parents could not remember the actual number of blood transfusions a child had taken before attending BMC, thus influencing our final results. Availability of commercial red cells is a problem because there are few Haematological laboratories worldwide that can manufacture these red cells to cover the demands.

Our antibody screening/identification approach might be prohibitive for extensive antibody screening in institutions with larger samples requiring shorter turnaround time because it is labour intensive and time-consuming. Other methods like Gel, SPRCA, or even automated methods can be used as these are faster, thus reducing laboratory turnaround time.

### Recommendations

Due to the common occurrence of alloantibodies and its detection within multiply transfused SCD patients in BMC, the BMC blood bank and Zonal National Blood Transfusion Centre should obtain facilities and expertise that will allow for extensive phenotypic blood typing and matching to minimise the development of alloantibodies and effectively minimize risks of delayed haemolytic transfusion reaction in SCD and chronically transfused patients.

### Conclusion

The rate of alloimmunisation in multiply transfused SCD patients was 8.5% and higher than other studies from East and West Africa. Thus, there is the need for extensive red blood cell screening and matching to minimize alloimmunisation and risk of delayed haemolytic transfusion reaction, particularly in SCD and chronically transfused patients.
